# *Notes from the Field*: Travel-Associated Melioidosis and Resulting Laboratory Exposures — United States, 2016

**DOI:** 10.15585/mmwr.mm6637a8

**Published:** 2017-09-22

**Authors:** Patrick K. Mitchell, Colin Campbell, Martha P. Montgomery, Julie Paoline, Christopher Wilbur, Leah Posivak-Khouly, Kristin Garafalo, Mindy Elrod, Lindy Liu, Andre Weltman

**Affiliations:** ^1^Epidemic Intelligence Service, CDC; ^2^Pennsylvania Department of Health; ^3^New Jersey Department of Health; ^4^Ohio Department of Health; ^5^Montgomery County Health Department, Norristown, Pennsylvania; ^6^Pediatric Infectious Diseases, Children’s Hospital of Philadelphia, Philadelphia, Pennsylvania; ^7^Division Of High-Consequence Pathogens and Pathology, CDC.

In mid-July 2016, a Pennsylvania resident aged 15 years who had recently returned from Thailand was treated by a pediatrician for sore throat, fever, and bilateral thigh abscesses at the sites of mosquito bites (Figure). She had traveled to northeast Thailand with nine other teens as part of an 18-day service-oriented trip run by an Ohio-based youth tour company that arranges travel to Thailand for approximately 500 persons annually. This trip included construction and agricultural activities and recreational mud exposures. The patient subsequently developed right inguinal lymphadenopathy and worsening abscesses, which prompted specimen collection for culture on August 25. This specimen was sent to a commercial laboratory in New Jersey, which identified *Burkholderia pseudomallei*, the causative organism of melioidosis, on August 30. The patient did not experience pneumonia or bacteremia, and recovered fully after 2 weeks of intensive therapy with parenteral ceftazidime and a 6-month outpatient course of eradication therapy with doxycycline.

**FIGURE:**
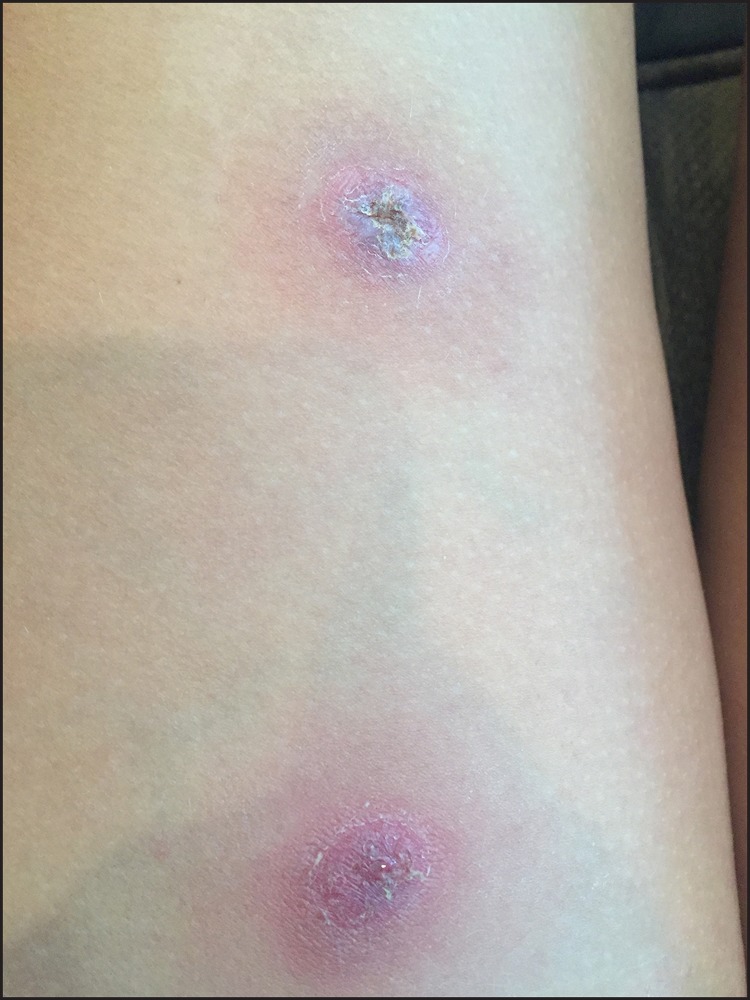
Thigh abscesses at the sites of mosquito bites in a Pennsylvania resident aged 15 years who had recently returned from Thailand, July 2016 Photo/patient (used with permission, name withheld for confidentiality) * Photo taken 7 weeks after onset.

Melioidosis has variable, nonspecific presentation, which can include cutaneous infection, pneumonia, bacteremia, septicemia, and other manifestations, after an incubation period of 1–21 days, although longer incubations of months or years have been reported (*1*,*2*). It is typically acquired from direct contact with soil or water contaminated with *B. pseudomallei*, which is highly endemic in northeast Thailand (*2*). Interviews with a tour company official revealed communication gaps regarding destination-specific health risks. With input from the Ohio and Pennsylvania Departments of Health, the tour company distributed a letter to participants and staff members who were on the patient’s trip, alerting them to melioidosis symptoms and exposure possibilities. No other trip participants responded to the letter to report symptoms. The tour company was advised to include CDC Yellow Book (*3*) resources in its predeparture materials for clients.

*B. pseudomallei* is not reportable in Pennsylvania, but is listed as a Tier 1 select agent, indicating its potential to pose a serious health threat (*4*). Although rare, laboratory acquisition of melioidosis through unknowing exposure to *B. pseudomallei* has been documented (*4*,*5*). Exposures for employees of the New Jersey commercial laboratory were categorized and managed according to published guidelines (*4*). Among 41 laboratory technologists assessed, serologic testing and symptom self-monitoring was recommended for two technologists who were exposed to aerosols while manipulating the culture outside of a biologic safety cabinet, and two who had predisposing medical conditions (diabetes [one] and long-term steroid use [one]) and were present in the laboratory during the aerosol-generating procedures. The two technologists handling the culture were also prescribed trimethoprim-sulfamethoxazole for antibiotic prophylaxis. One technologist developed fever, cough, and rash and was temporarily excluded from work. This was diagnosed as an adverse reaction to trimethoprim-sulfamethoxazole and resolved after switching to doxycycline. No melioidosis cases were identified among exposed laboratory technologists.

Because only zero to five cases of melioidosis are identified annually in the United States and the disease has nonspecific and possibly delayed symptoms, it might not initially be suspected as a diagnosis (*1*,*4*,*6*). When patient travel history is compatible with *B. pseudomallei* exposure, clinicians should have a higher index of suspicion and share this suspicion with laboratory personnel to reduce exposure risk. Persons on service-oriented trips might be at higher risk for acquiring melioidosis than a typical traveler because of the potential for quasi-occupational exposures such as construction and farm work. Travelers should be advised to seek information about the particular health risks associated with their destinations and planned activities, and should share this information with health care providers if symptoms develop. Travel organizers should also be informed of the health risks related to the destinations they serve and types of trips they offer.
